# Summary of taxonomy changes ratified by the International Committee on Taxonomy of Viruses from the Plant Viruses Subcommittee, 2025

**DOI:** 10.1099/jgv.0.002114

**Published:** 2025-07-25

**Authors:** Luisa Rubino, Peter Abrahamian, Wenxia An, Miguel A. Aranda, José T. Ascencio-Ibañez, Nicolas Bejerman, Arnaud G. Blouin, Thierry Candresse, Tomas Canto, Mengji Cao, John P. Carr, Won Kyong Cho, Fiona Constable, Indranil Dasgupta, Humberto Debat, Ralf G. Dietzgen, Michele Digiaro, Livia Donaire, Toufic Elbeaino, Denis Fargette, Fiona Filardo, Matthias G. Fischer, Nuria Fontdevila, Adrian Fox, Juliana Freitas-Astua, Marc Fuchs, Andrew D.W. Geering, Mahan Ghafari, Anders Hafrén, John Hammond, Rosemarie Hammond, Beata Hasiów-Jaroszewska, Eugenie Hebrard, Carmen Hernández, Jean-Michel Hily, Ahmed Hosseini, Roger Hull, Alice K. Inoue-Nagata, Ramon Jordan, Hideki Kondo, Jan F. Kreuze, Mart Krupovic, Kenji Kubota, Jens H. Kuhn, Scott Leisner, Jean-Michel Lett, Chengyu Li, Fan Li, Jun Min Li, Paola M. López-Lambertini, Juan J. Lopez-Moya, Francois Maclot, Kristiina Mäkinen, Darren Martin, Sebastien Massart, W. Allen Miller, Musa Mohammadi, Dimitre Mollov, Emmanuelle Muller, Tatsuya Nagata, Jesús Navas-Castillo, Yutaro Neriya, Francisco M. Ochoa-Corona, Kazusato Ohshima, Vicente Pallás, Hanu Pappu, Karel Petrzik, Mikhail Pooggin, Maria Isabella Prigigallo, Pedro L. Ramos-González, Simone Ribeiro, Katja R. Richert-Pöggeler, Philippe Roumagnac, Avijit Roy, Sead Sabanadzovic, Dana Šafářová, Pasquale Saldarelli, Hélène Sanfaçon, Cecilia Sarmiento, Takahide Sasaya, Kay Scheets, Willem E.W. Schravesande, Susan Seal, Yoshifumi Shimomoto, Merike Sõmera, Livia Stavolone, Lucy R. Stewart, Pierre-Yves Teycheney, John E. Thomas, Jeremy R. Thompson, Antonio Tiberini, Yasuhiro Tomitaka, Ioannis Tzanetakis, Marie Umber, Cica Urbino, Harrold A. van den Burg, René A.A. Van der Vlugt, Arvind Varsani, Adriaan Verhage, Dan Villamor, Susanne von Bargen, Peter J. Walker, Thierry Wetzel, Anna E. Whitfield, Stephen J. Wylie, Caixia Yang, F. Murilo Zerbini, Song Zhang, ICTV Taxonomy Summary Consortium, E.M. Adriaenssens

**Affiliations:** 1 Istituto per la Protezione Sostenibile delle Piante, CNR, Bari, Italy; 2 USDA-ARS, BARC, National Germplasm Resources Laboratory, Beltsville, MD, USA; 3 Liaoning Key Laboratory of Urban Integrated Pest Management and Ecological Security, Shenyang University, Dadong, Shenyang, Liaoning, PR China; 4 Centro de Edafología y Biología Aplicada del Segura-CSIC, Murcia, Spain; 5 Department of Molecular and Structural Biochemistry, North Carolina State University, Raleigh, NC, USA; 6 Unidad de Fitopatología y Modelización Agrícola (UFYMA) INTA-CONICET, Buenos Aires, Argentina; 7 Plant Protection Department, Agroscope, Nyon, Switzerland; 8 UMR 1332 Biologie du Fruit et Pathologie, University of Bordeaux, INRAE, Domaine de la Grande Ferrade, 71 Avenue Edouard Bourlaux, CS 20032, 33882 Villenave d'Ornon Cedex, France; 9 Margarita Salas Center for Biological Research (CIB-CSIC) Spanish Council for Scientific Research (CSIC), Madrid, Spain; 10 National Citrus Engineering and Technology Research Center, Integrative Science Center of Germplasm Creation in Western China (CHONGQING) Science City, Citrus Research Institute, Southwest University, Beibei, Chongqing, PR China; 11 Department of Plant Sciences, University of Cambridge, Cambridge, UK; 12 Agriculture and Life Sciences Research Institute, Kangwon National University, Chuncheon, Republic of Korea; 13 Agriculture Victoria Research, Department of Energy, Environment and Climate Action and School of Applied Systems Biology, La Trobe University, Bundoora, Australia; 14 University of Delhi South Campus, Benito Juarez Road, New Delhi, India; 15 Queensland Alliance for Agriculture and Food Innovation, The University of Queensland, St Lucia QLD, Australia; 16 CIHEAM, Istituto Agronomico Mediterraneo of Bari, Via Ceglie 9, Valenzano (BA), Italy; 17 Virus South Data, CDV 230980, La Mure, France; 18 Queensland Department of Primary Industries, Brisbane, Australia; 19 Max Planck Institute for Marine Microbiology, Bremen, Germany; 20 Fera Science Ltd (Fera), York Biotech Campus, Sand Hutton, York, UK; 21 Embrapa Cassava and Fruits, Brazilian Agricultural Research Corporation, Cruz das Almas, Brazil; 22 Plant Pathology, Cornell University, Geneva, NY, USA; 23 Department of Biology, University of Oxford, Oxford, UK; 24 Swedish University of Agriculture, Almas Allé 5, Uppsala, Sweden; 25 USDA-ARS, USNA, Floral and Nursery Plants Research Unit, Beltsville, MD, USA; 26 USDA-ARS, BARC, Molecular Plant Pathology Laboratory, Beltsville, MD, USA; 27 Institute of Plant Protection-NRI, ul. Wł. Węgorka 20, Poznań, Poland; 28 PHIM Plant Health Institute, University of Montpellier, INRAE, CIRAD, IRD, Institute Agro, Montpellier, France; 29 Instituto de Biología Molecular y Celular de Plantas (IBMCP), Universitat Politècnica de Valencia-CSIC, Valencia, Spain; 30 Institut Français de la Vigne et du Vin, Le Grau du Roi, France; 31 Vali-e-Asr University of Rafsanjan, Department of Plant Protection, Rafsanjan, Iran; 32 Retired from John Innes Centre, Norwich, UK; 33 Embrapa Hortaliças, Brasília, DF, Brazil; 34 Institute of Plant Science and Resources, Okayama University, Kurashiki, Japan; 35 International Potato Center (CIP), Lima, Peru; 36 Institut Pasteur, Université Paris Cité, CNRS UMR6047, Archaeal Virology Unit, Paris, France; 37 Institute for Plant Protection, NARO, 2-1-18, Kannondai, Tsukuba, Japan; 38 Integrated Research Facility at Fort Detrick, National Institute of Allergy and Infectious Diseases, National Institutes of Health, Fort Detrick, Frederick, MD, USA; 39 Department of Biological Sciences, University of Toledo, Toledo, OH, USA; 40 CIRAD, UMR PVBMT, Saint Pierre, La Réunion, France; 41 State Key Laboratory for Conservation and Utilization of Bio-Resources in Yunnan, Yunnan Agricultural University, Kunming, PR China; 42 Institute of Plant Virology, Ningbo University, Ningbo, PR China; 43 Instituto de Patología Vegetal (IPAVE), INTA, Unidad de Fitopatología y Modelización Agrícola (UFYMA) INTA-CONICET, Córdoba, Argentina; 44 Centre for Research in Agricultural Genomics, CRAG (CSIC-IRTA-UAB-UB), Barcelona, Spain; 45 Department of Agricultural Sciences, University of Helsinki, Helsinki, Finland; 46 Institute of Infectious Disease and Molecular Medicine, University of Cape Town, Cape Town, South Africa; 47 Plant Pathology Laboratory, TERRA Gembloux Agro-Bio Tech, University of Liege, Liege, Belgium; 48 Department of Plant Pathology, Entomology and Microbiology, Iowa State University, Ames, IA, USA; 49 Department of Plant Protection, Gorgan University of Agricultural Sciences and Natural Resources, Gorgan, Iran; 50 USDA-APHIS, Plant Protection and Quarantine, 4700 River Road, Riverdale, MD, USA; 51 CIRAD, AGAP Institut; AGAP Institut, University of Montpellier; CIRAD, INRAE, Institut Agro, 34398 Montpellier, France; 52 Instituto de Ciências Biológicas, Universidade de Brasília, Brasília, Brazil; 53 Instituto de Hortofruticultura Subtropical y Mediterránea “La Mayora” (IHSM-UMA-CSIC), Consejo Superior de Investigaciones Científicas, Algarrobo-Costa, Málaga, Spain; 54 Utsunomiya University, Utsunomiya, Japan; 55 Oklahoma State University, Institute for Biosecurity & Microbial Forensics, 127 NRC Stillwater, OK, USA; 56 Saga University, Saga, Japan; 57 Department of Plant Pathology, Washington State University, Pullman, WA, USA; 58 Institute of Plant Molecular Biology, České Budějovice, Czech Republic; 59 Applied Molecular Biology Laboratory, Instituto Biológico de São Paulo, São Paulo, Brazil; 60 Embrapa Recursos Genéticos e Biotecnologia, Brasília, Brazil; 61 Julius Kühn Institute, Federal Research Centre for Cultivated Plants, Institute for Epidemiology and Pathogen Diagnostics, Braunschweig, Germany; 62 CIRAD, UMR PHIM, Montpellier, France; 63 Department of Agricultural Science and Plant Protection, Mississippi State University, Mississippi State, MS, USA; 64 Department of Cell Biology and Genetics, Faculty of Science, Palacký University Olomouc, Olomouc, Czech Republic; 65 Summerland Research and Development Centre, Agriculture and Agri-Food Canada, Summerland, Canada; 66 Department of Chemistry and Biotechnology, Tallinn University of Technology, Tallinn, Estonia; 67 Strategic Planning Headquarters, NARO, 3-1-1 Kannondai, Tsukuba, Japan; 68 Department of Plant Pathology, Ecology and Evolution, Oklahoma State University, Stillwater, OK, USA; 69 Molecular Plant Pathology, University of Amsterdam, Amsterdam, Netherlands; 70 Natural Resources Institute, University of Greenwich, Central Avenue, Chatham Maritime, Kent, UK; 71 Kochi Agricultural Research Center, Nankoku, Japan; 72 Currently unaffiliated; 73 CIRAD, UMR PVBMT & UMR PVBMT, Université de la Réunion, F-97410 Saint Pierre de La Réunion, France; 74 Plant Health and Environment Laboratory, 231 Morrin Rd, St. Johns, Auckland, New Zealand; 75 Council for Agricultural Research and Economics, Research Centre for Plant Protection and Certification, Rome, Italy; 76 Department of Entomology and Plant Pathology, Division of Agriculture, University of Arkansas System, Fayetteville, AR, USA; 77 INRAE, UR ASTRO, Domaine de Duclos, Petit Bourg, France; 78 Wageningen University and Research, Wageningen, Netherlands; 79 The Biodesign Center for Fundamental and Applied Microbiomics, Center for Evolution and Medicine, School of Life Sciences, Arizona State University, Tempe, AZ, USA; 80 Rijk Zwaan Breeding B.V., De Lier, Netherlands; 81 Humboldt-Universität zu Berlin, Thaer-Institute of Agricultural and Horticultural Sciences, Unter den Linden 6, Berlin, Germany; 82 The University of Queensland, St Lucia, Australia; 83 Dienstleistungszentrum Ländlicher Raum Rheinpfalz, Breitenweg 71, Neustadt an der Weinstrasse, Germany; 84 North Carolina State University, Raleigh, NC, USA; 85 Food Futures Institute, Murdoch University, 90 South Street, Perth, Australia; 86 Dep. de Fitopatologia/BIOAGRO, Universidade Federal de Viçosa, Viçosa, Brazil

**Keywords:** *Actinidivirus*, *Ahlum waterborne virus*, *Albetovirus alphatabaci*, *Albetovirus betatabaci*, *Albetovirus gammatabaci*, *Allexivirus rehmanniae*, *Alphacytorhabdovirus*, *Alphacytorhabdovirus actinidiae*, *Alphacytorhabdovirus allii*, *Alphacytorhabdovirus alphaartemisiae*, *Alphacytorhabdovirus alphachysanthemi*, *Alphacytorhabdovirus alphafragariae*, *Alphacytorhabdovirus alphamedicagonis*, *Alphacytorhabdovirus alphapogostemi*, *Alphacytorhabdovirus alphaprimulae*, *Alphacytorhabdovirus alpharubi*, *Alphacytorhabdovirus alphatrifolii*, *Alphacytorhabdovirus alphawuhaninsectum*, *Alphacytorhabdovirus arctii*, *Alphacytorhabdovirus asclepiadis*, *Alphacytorhabdovirus baccharis*, *Alphacytorhabdovirus bacopae*, *Alphacytorhabdovirus betaartemisiae*, *Alphacytorhabdovirus betachrysanthemi*, *Alphacytorhabdovirus betafragariae*, *Alphacytorhabdovirus betamedicagonis*, *Alphacytorhabdovirus betapogostemi*, *Alphacytorhabdovirus betaprimulae*, *Alphacytorhabdovirus betarubi*, *Alphacytorhabdovirus betatrifolii*, *Alphacytorhabdovirus betawuhaninsectum*, *Alphacytorhabdovirus brassicicolae*, *Alphacytorhabdovirus cardaminis*, *Alphacytorhabdovirus chelidonii*, *Alphacytorhabdovirus cnidii*, *Alphacytorhabdovirus conopholis*, *Alphacytorhabdovirus coriandri*, *Alphacytorhabdovirus cynarae*, *Alphacytorhabdovirus daphnis*, *Alphacytorhabdovirus deltapogostemi*, *Alphacytorhabdovirus euphorbiae*, *Alphacytorhabdovirus fici*, *Alphacytorhabdovirus fragariarugosus*, *Alphacytorhabdovirus gammaartemisiae*, *Alphacytorhabdovirus gammapogostemi*, *Alphacytorhabdovirus gammawuhaninsectum*, *Alphacytorhabdovirus gei*, *Alphacytorhabdovirus glehniae*, *Alphacytorhabdovirus hederae*, *Alphacytorhabdovirus hyptisis*, *Alphacytorhabdovirus ilicis*, *Alphacytorhabdovirus kenyatuberosum*, *Alphacytorhabdovirus lactucanecante*, *Alphacytorhabdovirus lactucamaculante*, *Alphacytorhabdovirus lycopersici*, *Alphacytorhabdovirus menthae*, *Alphacytorhabdovirus morindae*, *Alphacytorhabdovirus nymphaeae*, *Alphacytorhabdovirus ocimi*, *Alphacytorhabdovirus paludis*, *Alphacytorhabdovirus pastinacae*, *Alphacytorhabdovirus pelargonii*, *Alphacytorhabdovirus persimmon*, *Alphacytorhabdovirus phyllostachysis*, *Alphacytorhabdovirus pinelliae*, *Alphacytorhabdovirus plumagonis*, *Alphacytorhabdovirus querci*, *Alphacytorhabdovirus ribes*, *Alphacytorhabdovirus rosae*, *Alphacytorhabdovirus sambuci*, *Alphacytorhabdovirus scutellariae*, *Alphacytorhabdovirus taraxaci*, *Alphacytorhabdovirus tolmieae*, *Alphacytorhabdovirus trichosanthei*, *Alphacytorhabdovirus tritici*, *Alphacytorhabdovirus utriculariae*, *Alphacytorhabdovirus wurfbainiae*, *Alphacytorhabdovirus zeae*, *Alphanucleorhabdovirus babaci*, *Aumaivirus maidis*, *Badnavirus fatsiae*, *Badnavirus tetainflatheobromae*, *Badnavirus ziziphi*, *Banana virus X*, *Bean mild mosaic virus*, *Begomovirus alceacrispi*, *Begomovirus caboniensis*, *Begomovirus cajani*, *Begomovirus chuxiongense*, *Begomovirus flavintervenae*, *Begomovirus galii*, *Begomovirus hortuscrotoni*, *Begomovirus hyptidis*, *Begomovirus jatrophagunturense*, *Begomovirus muntiflavi*, *Begomovirus myanmarense*, *Begomovirus puerense*, *Begomovirus pyrenacanthae*, *Begomovirus sidaflavitessellati*, *Begomovirus solanumaureusreti*, *Begomovirus solanumdistorsionis*, *Begomovirus solanumflavusreti*, *Begomovirus whitaniae*, *Betacytorhabdovirus*, *Betacytorhabdovirus alphabetulae*, *Betacytorhabdovirus alphacucurbitae*, *Betacytorhabdovirus alpharosae*, *Betacytorhabdovirus alphaturbaesoli*, *Betacytorhabdovirus alphazanthoxyli*, *Betacytorhabdovirus anthurii*, *Betacytorhabdovirus aristolochiae*, *Betacytorhabdovirus artemisiae*, *Betacytorhabdovirus begoniae*, *Betacytorhabdovirus bemisiae*, *Betacytorhabdovirus betabetulae*, *Betacytorhabdovirus betacucurbitae*, *Betacytorhabdovirus betarosae*, *Betacytorhabdovirus betaturbaesoli*, *Betacytorhabdovirus betazanthoxyli*, *Betacytorhabdovirus bouteloae*, *Betacytorhabdovirus broussonetiae*, *Betacytorhabdovirus caricae*, *Betacytorhabdovirus chrysanthemi*, *Betacytorhabdovirus colocasiae*, *Betacytorhabdovirus coryli*, *Betacytorhabdovirus cypripedii*, *Betacytorhabdovirus dryobalanopis*, *Betacytorhabdovirus durionis*, *Betacytorhabdovirus flaviyerbamate*, *Betacytorhabdovirus gammazanthoxyli*, *Betacytorhabdovirus gleditsiae*, *Betacytorhabdovirus glycinis*, *Betacytorhabdovirus glycyrrhizae*, *Betacytorhabdovirus gramineae*, *Betacytorhabdovirus hepaticae*, *Betacytorhabdovirus hordei*, *Betacytorhabdovirus howeae*, *Betacytorhabdovirus ipomoeae*, *Betacytorhabdovirus ixeris*, *Betacytorhabdovirus justiciae*, *Betacytorhabdovirus kobresiae*, *Betacytorhabdovirus leucadendri*, *Betacytorhabdovirus lycii*, *Betacytorhabdovirus mangonis*, *Betacytorhabdovirus maydis*, *Betacytorhabdovirus maysflavostriatis*, *Betacytorhabdovirus mori*, *Betacytorhabdovirus nitrariae*, *Betacytorhabdovirus oryzae*, *Betacytorhabdovirus panici*, *Betacytorhabdovirus passiflorae*, *Betacytorhabdovirus pentaphragmae*, *Betacytorhabdovirus phellodendri*, *Betacytorhabdovirus populi*, *Betacytorhabdovirus puerariae*, *Betacytorhabdovirus rudbeckiae*, *Betacytorhabdovirus schiedeae*, *Betacytorhabdovirus sesami*, *Betacytorhabdovirus sophorae*, *Betacytorhabdovirus tagetis*, *Betacytorhabdovirus tiliae*, *Betacytorhabdovirus trifolii*, *Betacytorhabdovirus viciae*, *Betacytorhabdovirus yerbamate*, *Betanucleorhabdovirus paridis*, *Bombyx mori latent virus*, *Botrexvirus duosclerotiniae*, *Botrexvirus unosclerotiniae*, *Capulavirus trifolii*, *Chenopodium necrosis virus*, *Cilevirus pistaciae*, *Citlodavirus apijamaicaense*, *Citlodavirus myricae*, *Cocksfoot mottle virus*, *Cucumber soil-borne virus*, *Cytorhabdovirus*, *Cytorhabdovirus brassicae*, *Cytorhabdovirus festucae*, *Cytorhabdovirus sonchi*, *Cytorhabdovirus tritici*, *Emaravirus clematis*, *Fabavirus betavitis*, *Fabavirus cirsii*, *Gammacytorhabdovirus*, *Gammacytorhabdovirus alphacuscutae*, *Gammacytorhabdovirus alphafraxini*, *Gammacytorhabdovirus apii*, *Gammacytorhabdovirus argyranthemi*, *Gammacytorhabdovirus betacuscutae*, *Gammacytorhabdovirus betafraxini*, *Gammacytorhabdovirus coptis*, *Gammacytorhabdovirus cypripedii*, *Gammacytorhabdovirus dauci*, *Gammacytorhabdovirus epipactis*, *Gammacytorhabdovirus gymnadeniae*, *Gammacytorhabdovirus heliospermae*, *Gammacytorhabdovirus hibisci*, *Gammacytorhabdovirus lonatis*, *Gammacytorhabdovirus lupinis*, *Gammacytorhabdovirus rhopalocnemis*, *Gammacytorhabdovirus silenis*, *Gammacytorhabdovirus trachyspermi*, *Higrevirus amurense*, *Higrevirus pistaciae*, *Ilarvirus ApNMV*, *Ilarvirus BabIV1*, *Ilarvirus TIV1*, *Ilarvirus ToNSV*, *Machlomovirus liegense*, *Maize aumaivirus 1*, *Mastrevirus bothriochloae*, *Mastrevirus brachypodiumprimi*, *Mastrevirus brachypodiumsecundi*, *Mastrevirus nomiae*, *Mastrevirus urochloareunionense*, *Mersevirus*, *Mersevirus boehmeriae*, *Mersevirus jujubae*, *Mersevirus mercurialis*, *Mersevirus paris*, *Nepovirus betaparis*, *Nepovirus mirae*, *Olpivirus lactucae*, *Orthotospovirus eustomae*, *Orthotospovirus fatsiae*, *Pamosaviridae*, *Panicum papanivirus 1*, *Papanivirus panici*, *Phragmivirus*, *Phragmivirus phragmii*, *Phragmivirus spartinae*, *Poinsettia mosaic virus*, *Potexvirus chaenostomae*, *Potexvirus ecsadenii*, *Potexvirus ecscaricae*, *Potexvirus ecshibisci*, *Potyvirus aconiti*, *Potyvirus alilii*, *Potyvirus catharanthiflavitessellati*, *Potyvirus crocitessellati*, *Potyvirus galanthi*, *Potyvirus parisflavitessellati*, *Potyvirus polygonatimaculae*, *Potyvirus puerariae*, *Ritunrivirus*, *Sadwavirus cattleyae*, *Sadwavirus chysanthemi*, *Sadwavirus gymnemae*, *Sobemovirus CFMV*, *Sobemovirus SMAMV*, *Sobemovirus smamv*, *Tobacco albetovirus 1*, *Tobacco albetovirus 2*, *Tobacco albetovirus 3*, *Tobacco virtovirus 1*, *Tombendovirales*, *Tomosaviridae*, *Tonesaviridae*, *Torradovirus arctii*, *Torradovirus nanorugosum*, *Torradovirus physalis*, *Trirhavirus*, *Trirhavirus alni*, *Trirhavirus chrysanthemi*, *Trirhavirus erysimi*, *Trirhavirus medicagonis*, *Trirhavirus picridis*, *Velarivirus gembloutense*, *Virtovirus tabaci*, *Waikavirus*, *Waikavirus ajugae*, *Waikavirus anacycli*, *Waikavirus betacamelliae*, *Waikavirus carotae*, *Waikavirus celtis*, *Waikavirus eleocharis*, *Waikavirus hirtae*, *Waikavirus juglandis*, *Waikavirus ligustici*, *Waikavirus mertensiae*, *Waikavirus pedicularis*, *Waikavirus pittospori*, *Waikavirus populi*, *Waikavirus primulae*, *Waikavirus querci*, *Waikavirus ranunculi*, *Waikavirus thapsiae*, *Waikavirus thymi*, *Waikavirus trifoccidentale*, *Waikavirus violae*, *Weddel waterborne virus*

## Abstract

In March 2025, following the annual International Committee on Taxonomy of Viruses (ICTV) ratification vote, newly proposed taxa were added to those under the mandate of the Plant Viruses Subcommittee. In brief, 1 new order, 3 new families, 6 new genera, 2 new subgenera and 206 new species were created. Some taxa were reorganized. Genus *Cytorhabdovirus* in the family *Rhabdoviridae* was abolished and its taxa were redistributed into three new genera *Alphacytorhabdovirus*, *Betacytorhabdovirus* and *Gammacytorhabdovirus*. Genus *Waikavirus* in the family *Secoviridae* was reorganized into two subgenera (*Actinidivirus* and *Ritunrivirus*). One family and four previously unaffiliated genera were moved to the newly established order *Tombendovirales*. Twelve species not assigned to a genus were abolished. To comply with the ICTV mandate of a binomial format for virus species, eight species were renamed. Demarcation criteria in the absence of biological information were defined in the genus *Ilarvirus* (family *Bromoviridae*). This article presents the updated taxonomy put forth by the Plant Viruses Subcommittee and ratified by the ICTV.

## Introduction

The Plant Viruses Subcommittee of the International Committee on Taxonomy of Viruses (ICTV) deals with the taxonomic classification of most viruses infecting or isolated from plants [1]. It consists of 22 Study Groups, composed of more than 180 members, covering 27 virus families.

Viruses, viroids, satellite viruses and satellite nucleic acids originally isolated from plants have been classified into 2,392 species belonging to 247 genera in 55 families, 25 orders, 16 classes, 7 phyla, 3 kingdoms and 2 realms [2]. Following the 2025 ICTV ratification vote, these taxa were expanded by creating 1 new order, 3 new families, 6 new genera, 2 new subgenera and 206 new species.

To comply with the ICTV mandate of a binomial format for virus species [3,4], species names will consist of the genus name followed by a Latinized or not Latinized (freeform) epithet [5,6]. The adoption of the binomial format for virus species names was completed by renaming eight species. Similarly, 12 species in the families *Rhabdoviridae* and *Tombusviridae* were abolished because they could not be assigned to a genus due to the lack of a genome sequence. Viruses in many of the new species were discovered by metagenomic analysis and were classified according to Simmonds *et al.* [7].

Some established taxa have been extensively reorganized. The family *Rhabdoviridae* has been expanded by the creation of one new genus, whereas the genus *Cytorhabdovirus* was abolished and replaced with three new genera *Alphacytorhabdovirus*, *Betacytorhabdovirus* and *Gammacytorhabdovirus*, representing distinct evolutionary lineages. In the family *Secoviridae*, one new genus was created and the established genus *Waikavirus* was reorganized into two subgenera. The establishment of the new order *Tombendovirales* allowed the placement of four previously unaffiliated genera of plant satellite viruses into four families based on high-resolution capsid structure studies rather than sequence identity.

The classification of viruses discovered by metagenomics prompted some refinement of demarcation criteria in the absence of biological information. For instance, species demarcation criteria based on ‘serology, host range and sequence similarity’ in the genus *Ilarvirus*, family *Bromoviridae*, have been refined to require less than 85% amino acid identity for the complete RNA2-encoded 2a protein.

All of these changes contribute to the advancement of virus taxonomy. To disseminate taxonomy decisions, the annual publication of summaries of all taxonomy proposals from each ICTV Subcommittee will provide a compendium of the taxonomy changes [8]. Therefore, the updated taxonomy from the Plant Viruses Subcommittee as now accepted by the ICTV is summarized in this article. It should be noted that the article does not necessarily cover all changes in the taxonomy of plant viruses, and consultation of reports from the Fungal and Protist Viruses Subcommittee [9] and the Animal dsRNA and ssRNA(−) Viruses Subcommittee [10] may be needed. A file including all the Tables of taxonomic changes below is available as a supplementary file to this article.

## Main Text

## Contents

2024.001P.Fimoviridae_1nsp
2024.002P.Alphaflexiviridae_7nsp
2024.003P.Tospoviridae_2nsp
2024.004P.Konkoviridae_1nsp
2024.005P.Caulimoviridae_3nsp
2024.006P.Kitaviridae_3nsp
2024.007P.Geminiviridae_Capulavirus_1nsp
2024.008P.Geminiviridae_Citlodavirus_2nsp
2024.009P.Geminiviridae_Mastrevirus_5nsp
2024.010P.Geminiviridae_Begomovirus_18nsp
2024.011P.Bromoviridae_4nsp
2024.012P.Potyviridae_1ng_10nsp
2024.013P.Secoviridae_1ng_2nsg_34nsp
2024.014P.Rhabdoviridae_2nsp
2024.015P.Rhabdoviridae_Cytorhabdovirus_splitgen
2024.016P.Rhabdoviridae_1ngen_5nsp
2024.017P.Tombusviridae_abolishsp
2024.018P.Tombusviridae_1nsp
2024.019P.Closteroviridae_1nsp
2024.021P.Riboviria_1nord
2024.022P.Betaflexiviridae_abolishsp
2024.023P.Solemoviridae_rename_sp
2024.024P.Tymoviridae_abolish_sp

## 2024.001P.Fimoviridae_1nsp

**Title:** Create *Emaravirus clematis* as a new species in the genus *Emaravirus*, family *Fimoviridae*

**Authors:** Yang C., An W., Li C., Zhang S., Cao M., Digiaro M. (digiaro@iamb.it), Elbeaino T., Kubota K., Ochoa Corona F.M., von Bargen S.

**Summary**

**Taxonomic rank(s) affected**
Species
**Description of current taxonomy**
The family *Fimoviridae* currently includes 32 virus species in the genus *Emaravirus*.
**Proposed taxonomic change(s)**
Add one (1) new virus species to genus *Emaravirus* within the family *Fimoviridae*.
**Justification**
The creation of the new species *Emaravirus clematis* in the genus *Emaravirus*, family *Fimoviridae*, is proposed to accommodate Clematis yellow mottle-associated virus, identified in China on *Clematis brevicaudata* DC. The virus assigned to the new species consists of a fully sequenced five-segmented, linear, single-stranded (ss), negative-sense RNA genome (of which two RNA3s encode the nucleocapsid protein), which shows features common to homologous RNAs of viruses assigned to other *Emaravirus* species but from which they differ significantly in nucleotide and amino acid sequences.
**Submitted:** 30/04/24; **Revised**: 16/09/24

**Table 1** *Fimoviridae*, 1 new taxon*

**Table:** 

Operation	Rank	New taxon name	Virus name	Exemplar
New taxon	Species	*Emaravirus clematis*	Clematis yellow mottle-associated virus	RNA1: OP807964; RNA2: OP807965; RNA3a: OP807966; RNA3b: OP807967; RNA4: OP807968

*Source/full text: https://ictv.global/ictv/proposals/2024.001P.Fimoviridae_1nsp.zip.

## 2024.002P.Alphaflexiviridae_7nsp

**Title:** Create seven new species in the family *Alphaflexiviridae*

**Authors:** Abrahamian P., Donaire L., Candresse T., Fox A., Hammond J., Hasiów-Jaroszewska B., Kreuze J., Rubino L., Aranda M.A. (m.aranda@cebas.csic.es)

**Summary**

**Taxonomic rank(s) affected**
Species
**Description of current taxonomy**
The family *Alphaflexiviridae* currently includes 65 virus species in genera *Allexivirus* (13), *Botrexvirus* (1), *Lolavirus* (1), *Platypuvirus* (1), *Potexvirus* (48) and *Sclerodarnavirus* (1).
**Proposed taxonomic change(s)**
Add seven (7) new virus species to genera *Allexivirus* (1), *Botrexvirus* (2) and *Potexvirus* (4) within the family *Alphaflexiviridae*.
**Justification**
Throughout the family, isolates of viruses assigned to different species have less than 72% nucleotide identity (or 80% amino acid identity) among their respective coat protein or polymerase genes (or proteins). Viruses from different genera usually have <45% nucleotide identity in these genes. The nucleotide or amino acid sequences of viruses belonging to the seven newly proposed species fit well within these demarcation criteria.
**Submitted**: 14/06/24

**Table 2** *Alphaflexiviridae*, 7 new taxa*

**Table:** 

Operation	Rank	New taxon name	Virus name	Exemplar
New taxon	Species	*Allexivirus rehmanniae*	Rehmannia allexivirus	PP097219
New taxon	Species	*Botrexvirus unosclerotiniae*	Sclerotinia sclerotiorum alphaflexivirus 1	ON993219
New taxon	Species	*Botrexvirus duosclerotiniae*	Sclerotinia sclerotiorum alphaflexivirus 2	OQ865609
New taxon	Species	*Potexvirus ecsadenii*	Adenium obesum virus X	OR039325
New taxon	Species	*Potexvirus chaenostomae*	Chaenostoma potexvirus	OL979628
New taxon	Species	*Potexvirus ecshibisci*	Hibiscus virus X	PP115950
New taxon	Species	*Potexvirus ecscaricae*	papaya virus X	MN265368

*Source/full text: https://ictv.global/ictv/proposals/2024.002P.Alphaflexiviridae_7nsp.zip.

## 2024.003P.Tospoviridae_2nsp

**Title:** Create two new species in the genus *Orthotospovirus* (*Elliovirales*: *Tospoviridae*)

**Authors:** Tomitaka Y., Shimomoto Y., Sasaya T. (tsasaya@affrc.go.jp)

**Summary**

**Taxonomic rank(s) affected**
Species
**Description of current taxonomy**
The family *Tospoviridae* currently includes 28 virus species in the genus *Orthotospovirus*.
**Proposed taxonomic change(s)**
Add two (2) new virus species to genus *Orthotospovirus* within the family *Tospoviridae*.
**Justification**
It is proposed that two (2) newly discovered tospovirids be classified into new species in the genus *Orthotospovirus* on the basis of species demarcation criteria in the amino acid sequence of the RNA-directed RNA polymerase and nucleocapsid protein.
**Submitted:** 13/06/24

**Table 3** *Tospoviridae*, 2 new taxa*

**Table:** 

Operation	Rank	New taxon name	Virus name	Exemplar
New taxon	Species	*Orthotospovirus eustomae*	lisianthus necrotic ringspot virus	MF469045; MF469046; MF469047
New taxon	Species	*Orthotospovirus fatsiae*	Fatsia japonica ringspot-associated virus	LC626335; LC626336; LC626337

*Source/full text: https://ictv.global/ictv/proposals/2024.003P.Tospoviridae_2nsp.zip.

## 2024.004P.Konkoviridae_1nsp

**Title:** Create one new species in the genus *Olpivirus* (*Hareavirales*: *Konkoviridae*)

**Authors:** Neriya Y., Schravesande W.E.W., van den Burg H.A., Verhage A., Tomitaka Y., Sasaya T. (tsasaya@affrc.go.jp)

**Summary**

**Taxonomic rank(s) affected**
Species
**Description of current taxonomy**
The family *Konkoviridae* currently includes one virus species in the genus *Olpivirus*.
**Proposed taxonomic change(s)**
Add one (1) new virus species to genus *Olpivirus* within the family *Konkoviridae*.
**Justification**
It is proposed that one (1) newly discovered konkovirid be classified into a new species in the genus *Olpivirus* on the basis of a species demarcation criterion of <95% identity in the amino acid sequence of the RNA-directed RNA polymerase.
**Submitted:** 13/06/24; **Revised:** 07/10/24

**Table 4** *Konkoviridae*, 1 new taxon*

**Table:** 

Operation	Rank	New taxon name	Virus name	Exemplar
New taxon	Species	*Olpivirus lactucae*	Lactuca big vein associated phlebovirus	RNA1: OR610326; RNA2: OR610327; RNA3: OR610328; RNA4: OR610329

*Source/full text: https://ictv.global/ictv/proposals/2024.004P.Konkoviridae_1nsp.zip.

## 2024.005P.Caulimoviridae_3nsp

**Title:** Create three new species in the genus *Badnavirus* (*Ortervirales*: *Caulimoviridae*)

**Authors:** Umber M., Dasgupta I., Geering A.D.W., Hafrén A., Hull R., Kreuze J., Leisner S., Muller E., Pappu H., Pooggin M., Richert-Pöggeler K.R., Seal S., Stavolone L., Teycheney P.Y. (teycheney@cirad.fr)

**Summary**

**Taxonomic rank(s) affected**
Genus (*Badnavirus*)
**Description of current taxonomy**
The family *Caulimoviridae* [11] currently comprises 11 genera whose members share a similar genome organization. The molecular species demarcation criterion is <80% identity of nucleotide sequences in the reverse transcriptase/ribonuclease H region of the polymerase. Genus *Badnavirus* [12] currently includes 71 species and is the largest genus within the family *Caulimoviridae*.
**Proposed taxonomic change(s)**
Add three (3) new species to the genus *Badnavirus* (*Badnavirus fatsiae*, *Badnavirus tetainflatheobromae* and *Badnavirus ziziphi*).
**Justification**
Complete genomes of the exemplar members of the three proposed new species were sequenced and published recently. Their organizations are similar to those of other members of the genus *Badnavirus*. Phylogenetic analyses place them in this genus as distinct representatives of novel species.
**Submitted:** 14/06/2024

**Table 5** *Caulimoviridae*, 3 new taxa*

**Table:** 

Operation	Rank	New taxon name	Virus name	Exemplar
New taxon	Species	*Badnavirus fatsiae*	Fatsia badnavirus 1	OM540428
New taxon	Species	*Badnavirus tetainflatheobromae*	cacao swollen shoot Ghana T virus	MN179342
New taxon	Species	*Badnavirus ziziphi*	jujube badnavirus WS	OL739567

*Source/full text: https://ictv.global/ictv/proposals/2024.005P.Caulimoviridae_3nsp.zip.

## 2024.006P.Kitaviridae_3nsp

**Title:** Create a new species in the genus *Cilevirus* and two in the genus *Higrevirus*, family *Kitaviridae* (*Martellivirales*)

**Authors:** Li C., An W., Zhang S., Cao M., Yang C. (xueyang27@126.com), Mohammadi M., Hosseini A., Nasrollanejad S., Roy A., Freitas-Astua J., Tiberini A., Li J.M., Ramos-González P.L.

**Summary**

**Taxonomic rank(s) affected**
Species
**Description of current taxonomy**
Family *Kitaviridae*, order *Martellivirales*, includes plant-infecting viruses having linear single-stranded (ss) positive-sense (+) segmented RNA genomes. Viruses in this family are assigned to the genera *Cilevirus*, *Higrevirus* or *Blunervirus* [13,14].
**Proposed taxonomic change(s)**
Add three (3) new species to the family *Kitaviridae*, one (1) to the genus *Cilevirus* and two (2) to the genus *Higrevirus*.
**Justification**
The genomes of the three novel viruses show an arrangement that resembles that of kitavirids, and their core conserved proteins share relatively low amino acid sequence identities (<85%) with recognized members of the family *Kitaviridae*. In phylogenetic analyses, the three viruses group with characterized members of the genera *Cilevirus* and *Higrevirus*, but they are well-separated and supported by bootstrap values >95%. All new species meet the already established or the demarcation criteria defined in this proposal.
**Submitted:** 11/06/2024

**Table 6** *Kitaviridae*, 3 new taxa*

**Table:** 

Operation	Rank	New taxon name	Virus name	Exemplar
New taxon	Species	*Higrevirus amurense*	Phellodendron-associated higre-like virus	RNA1: OP324809; RNA2: OP324810; RNA3: OP324811
New taxon	Species	*Higrevirus pistaciae*	pistachio virus X	RNA1: MT334620; RNA2: MT334619; RNA3: MT334618
New taxon	Species	*Cilevirus pistaciae*	pistachio virus Y	RNA1: MT362606; RNA2: MT362605

*Source/full text: https://ictv.global/ictv/proposals/2024.006P.Kitaviridae_3nsp.zip.

## 2024.007P.Geminiviridae_Capulavirus_1nsp

**Title:** Create one new species in the genus *Capulavirus* (*Geplafuvirales*: *Geminiviridae*)

**Authors:** Roumagnac P. (philippe.roumagnac@cirad.fr), Ascencio-Ibanez J., Lett J.-M., López-Lambertini P.M., Martin D.P., Navas-Castillo J., Ribeiro S., Urbino C., Varsani A., Zerbini F.M.

**Summary**

**Taxonomic rank(s) affected**
Genus (*Capulavirus*)
**Description of current taxonomy**
*Monodnaviria*: *Shotokuvirae*: *Cressdnaviricota*: *Repensiviricetes*: *Geplafuvirales*: *Geminiviridae*: *Capulavirus*.
**Proposed taxonomic change(s)**
Add one (1) new species to the genus *Capulavirus* (*Capulavirus trifolii*).
**Justification**
Similar to members of the *Capulavirus* genus, members of the proposed new species *Capulavirus trifolii* have the virion-strand origin of replication nonanucleotide motif ‘TAATATTAC’ and show a typical capulavirus genome organization, with putative multiple overlapping short ORFs (V3 and V4) upstream of the coat protein gene that encode putative movement proteins. In addition, genome-wide pairwise analysis of the representative genomes of capulaviruses showed that viruses in the species *Capulavirus trifolii* share <78% identity with all representative genomes of capulaviruses. Because 78% nucleotide identity is the genome-wide species demarcation threshold for capulaviruses, it was concluded that *Capulavirus trifolii* represents a new species in the genus *Capulavirus*.
**Submitted:** 10/06/24

**Table 7** *Geminiviridae*, 1 new taxon*

**Table:** 

Operation	Rank	New taxon name	Virus name	Exemplar
New taxon	Species	*Capulavirus trifolii*	Trifolium virus 1	MW698813

*Source/full text: https://ictv.global/ictv/proposals/2024.007P.Geminiviridae_Capulavirus_1nsp.zip.

## 2024.008P.Geminiviridae_Citlodavirus_2nsp

**Title:** Create two new species in the genus *Citlodavirus* (*Geplafuvirales*: *Geminiviridae*)

**Authors:** Roumagnac P. (philippe.roumagnac@cirad.fr), Ascencio-Ibanez J., Lett J.-M., López-Lambertini P.M., Martin D.P., Navas-Castillo J., Ribeiro S., Urbino C., Varsani A., Zerbini F.M.

**Summary**

**Taxonomic rank(s) affected**
Genus (*Citlodavirus*)
**Description of current taxonomy**
*Monodnaviria*: *Shotokuvirae*: *Cressdnaviricota*: *Repensiviricetes*: *Geplafuvirales*: *Geminiviridae*: *Citlodavirus*.
**Proposed taxonomic change(s)**
Add two (2) new species to genus *Citlodavirus* (*Citlodavirus apijamaicaense* and *Citlodavirus myricae*).
**Justification**
Similar to members of the *Citlodavirus* genus, members of the proposed new species *Citlodavirus apijamaicaense* and *Citlodavirus myricae* have the virion-strand origin of replication nonanucleotide motif ‘TAA TAT TAC’, a relatively large genome (3,918 and 3,775 nt, respectively) and unique genome arrangements that, in both cases, include the putative *mp* gene (888 and 912 nt, respectively) that is similar in size to the *mp* gene in the DNA-B of bipartite begomoviruses. Genome-wide pairwise analysis of the representative genomes of citlodaviruses showed that viruses in the species *Citlodavirus apijamaicaense* and *Citlodavirus myricae* share <78% identity with all representative genomes of citlodaviruses and with each other. Since 78% nucleotide identity is the genome-wide species demarcation threshold for citlodaviruses, it has been concluded that *Citlodavirus apijamaicaense* and *Citlodavirus myricae* represent two new species in the genus *Citlodavirus*.
**Submitted:** 10/06/2024

**Table 8** *Geminiviridae*, 2 new taxa*

**Table:** 

Operation	Rank	New taxon name	Virus name	Exemplar
New taxon	Species	*Citlodavirus apijamaicaense*	apiscitlodal virus	PP467584
New taxon	Species	*Citlodavirus myricae*	Myrica rubra citlodavirus 1	OP374189

*Source/full text: https://ictv.global/ictv/proposals/2024.008P.Geminiviridae_Citlodavirus_2nsp.zip.

## 2024.009P.Geminiviridae_Mastrevirus_5nsp

**Title:** Establish five new species in the genus *Mastrevirus*

**Authors:** Varsani A. (Arvind.varsani@asu.edu), Martin D.P., Roumagnac P., Ascencio-Ibanez J., Lett J.-M., López-Lambertini P.M., Navas-Castillo J., Ribeiro S., Urbino C., Zerbini F.M.

**Summary**

**Taxonomic rank(s) affected**
Species
**Description of current taxonomy**
*Monodnaviria*: *Shotokuvirae*: *Cressdnaviricota*: *Repensiviricetes*: *Geplafuvirales*: *Geminiviridae*: *Mastrevirus*. Within the genus *Mastrevirus*, viruses are classified into species based on a 78% genome-wide pairwise identity threshold [15].
**Proposed taxonomic change(s)**
Add five (5) new species to classify a suite of new mastreviruses that have been identified over the last year or so.
**Justification**
The members of the five new proposed species in the genus *Mastrevirus* share <78% genome-wide pairwise identity with sequences of members of currently established mastrevirus species.
**Submitted:** 10/06/24

**Table 9** *Geminiviridae*, 5 new taxa*

**Table:** 

Operation	Rank	New taxon name	Virus name	Exemplar
New taxon	Species	*Mastrevirus urochloareunionense*	Urochloa decumbens associated virus	OQ451139
New taxon	Species	*Mastrevirus nomiae*	nomiamastrel virus	PP467585
New taxon	Species	*Mastrevirus brachypodiumprimi*	Brachypodium phoenicoides associated virus 1	OR596402
New taxon	Species	*Mastrevirus bothriochloae*	Bothriochloa barbinodis associated virus	OR596403
New taxon	Species	*Mastrevirus brachypodiumsecundi*	Brachypodium phoenicoides associated virus 2	OR596405

*Source/full text:https://ictv.global/ictv/proposals/2024.009P.Geminiviridae_Mastrevirus_5nsp.zip.

## 2024.010P.Geminiviridae_Begomovirus_18nsp

**Title:** Create 18 new species in the genus *Begomovirus* (*Geplafuvirales*: *Geminiviridae*)

**Authors:** Zerbini F.M. (zerbini@ufv.br), Ascencio-Ibanez J., Lett J.M., Navas-Castillo J., Urbino C., López-Lambertini P.M., Martin D.P., Ribeiro S.G., Roumagnac P., Varsani A.

**Summary**

**Taxonomic rank(s) affected**
Species in the genus *Begomovirus*
**Description of current taxonomy**
*Monodnaviria*: *Shotokuvirae*: *Cressdnaviricota*: *Repensiviricetes*: *Geplafuvirales*: *Geminiviridae*: *Begomovirus*. Within the genus *Begomovirus*, viruses are classified into species based on a 91% genome-wide (or DNA-A in the case of bipartite viruses) pairwise identity threshold [16].
**Proposed taxonomic change(s)**
Creation of 18 new species to classify new begomoviruses that have been identified and described in the literature over the last 3 years.
**Justification**
All 18 proposed new species have <91% genome-wide (or DNA-A in the case of bipartite viruses) pairwise identity with sequences of members of currently established begomovirus species.
**Submitted:** 10/06/24

**Table 10** *Begomovirus*, 18 new taxa*

**Table:** 

Operation	Rank	New taxon name	Virus name	Exemplar
New taxon	Species	*Begomovirus chuxiongense*	tomato leaf curl Chuxiong virus	OR543988
New taxon	Species	*Begomovirus solanumaureusreti*	tomato golden net virus	MT214095
New taxon	Species	*Begomovirus solanumflavusreti*	tomato yellow net virus	MT214096
New taxon	Species	*Begomovirus whitaniae*	Withania leaf curl virus	OP617239
New taxon	Species	*Begomovirus cajani*	Cajanus scarabaeoides yellow mosaic virus	OM397101; OM397102
New taxon	Species	*Begomovirus hortuscrotoni*	garden croton enation leaf curl virus	MW816855; MW816857
New taxon	Species	*Begomovirus jatrophagunturense*	Jatropha leaf curl Guntur virus	MZ217773
New taxon	Species	*Begomovirus hyptidis*	Hyptis golden mosaic virus	ON073795; ON073796
New taxon	Species	*Begomovirus galii*	Galium leaf distortion virus	OL689630
New taxon	Species	*Begomovirus myanmarense*	tobacco curly shoot Myanmar virus	MK920410
New taxon	Species	*Begomovirus caboniensis*	Cnidoscolus mild mosaic virus	MZ465527; MZ465585
New taxon	Species	*Begomovirus pyrenacanthae*	Pyrenacantha yellow mosaic virus	MZ390982; MZ390984
New taxon	Species	*Begomovirus puerense*	tobacco leaf curl Puer virus	MZ465370
New taxon	Species	*Begomovirus solanumdistorsionis*	tomato mottle leaf distortion virus	MW561191; MW650837
New taxon	Species	*Begomovirus alceacrispi*	hollyhock vein yellowing virus	LK028571
New taxon	Species	*Begomovirus muntiflavi*	Muntingia yellow spot virus	MW032664; MW032665
New taxon	Species	*Begomovirus flavintervenae*	tomato interveinal yellowing virus	MW057360
New taxon	Species	*Begomovirus sidaflavitessellati*	Sida yellow mosaic Gujarat virus	KX513859

*Source/full text: https://ictv.global/ictv/proposals/2024.010P.Geminiviridae_Begomovirus_18nsp.zip.

## 2024.011P.Bromoviridae_4nsp

**Title:** Create four (4) new species in the genus *Ilarvirus* (*Martellivirales*: *Bromoviridae*)

**Authors:** Thompson J.R. (jeremy.thompson@mpi.govt.nz), Canto T., Carr J.P., Pallás V., Šafářová D.

**Summary**

**Taxonomic rank(s) affected**
Species
**Description of current taxonomy**
The family *Bromoviridae* currently includes 49 virus species in genera *Alfamovirus* (1), *Anulavirus* (4), *Bromovirus* (7), *Cucumovirus* (4), *Ilarvirus* (32) and *Oleavirus* (1).
**Proposed taxonomic change(s)**
Add four (4) new virus species to genus *Ilarvirus* within the family *Bromoviridae*.
**Justification**
This taxonomic proposal considers the recognition of four new virus species based on species demarcation criteria in the family *Bromoviridae*, genus *Ilarvirus* of ‘serology, host range and sequence similarity’. In the absence of biological information, it is proposed that a refinement of the ‘sequence similarity’ criterion be made to require that members of different species display <85% amino acid identity for the complete RNA2-encoded 2a protein.
**Submitted:** 10/06/24; **Revised**: 07/10/24

**Table 11** *Bromoviridae*, 4 new taxa*

**Table:** 

Operation	Rank	New taxon name	Virus name	Exemplar
New taxon	Species	*Ilarvirus ApNMV*	apple necrotic mosaic virus	LC108993; LC108994; LC108995
New taxon	Species	*Ilarvirus BabIV1*	babaco ilarvirus 1	OQ256238; OQ256239; OQ256240
New taxon	Species	*Ilarvirus TIV1*	tomato ilarvirus 1	OL472057; OL472058; OL472059
New taxon	Species	*Ilarvirus ToNSV*	tomato necrotic spot virus	MH780154; MH780155; MH780156

*Source/full text: https://ictv.global/ictv/proposals/2024.011P.Bromoviridae_4nsp.zip.

## 2024.012P.Potyviridae_1ng_10nsp

**Title:** Create 1 new genus (*Phragmivirus*) with 2 species and 8 new species in the genus *Potyvirus* (*Patatavirales*: *Potyviridae*)

**Authors:** Inoue-Nagata A.K. (alice.nagata@embrapa.br), Jordan R., Kreuze J.F., Li F., Lopez-Moya J.J., Mäkinen K., Ohshima K., Wylie S.J.

**Summary**

**Taxonomic rank(s) affected**
Genus within the family *Potyviridae* and species within the genus *Potyvirus* and the newly proposed genus *Phragmivirus*
**Description of current taxonomy**
According to the ICTV Report chapter on *Potyviridae*, 12 genera are differentiated by biological criteria, mainly transmission by specific vectors, and by molecular data, in which members of different genera are <46% identical in nucleotide sequence. Members of different species have complete ORF sequences that are generally <76% identical in nucleotide sequence and <82% identical in amino acid sequence. In considering the evidence for new species or genera in the family *Potyviridae*, the Study Group will evaluate each new case based on complete- or near-complete genome sequence(s) together with host and biological characteristics.
**Proposed taxonomic change(s)**
Create one (1) new genus (*Phragmivirus*), two (2) new species in the genus *Phragmivirus* (*Phragmivirus phragmii* and *Phragmivirus spartinae*) and eight (8) new species in the genus *Potyvirus* (*Potyvirus aconiti*, *Potyvirus puerariae*, *Potyvirus alilii*, *Potyvirus parisflavitessellati*, *Potyvirus catharanthiflavitessellati*, *Potyvirus polygonatimaculae*, *Potyvirus crocitessellati* and *Potyvirus galanthi*).
**Justification**
The genomes of the proposed members in the new genus *Phragmivirus* share sequence identity below the threshold for genus differentiation in the family *Potyviridae*; members of the proposed species have a genome strategy typical of members of genus *Phragmivirus* (two species) and *Potyvirus* (eight species), and their nucleotide and amino acid sequences are below the threshold for species demarcation criteria for the genera.
**Submitted:** 11/06/24; **Revised:** 21/09/24

**Table 12** *Potyviridae*, 11 new taxa*

**Table:** 

Operation	Rank	New taxon name	Virus name	Exemplar
New taxon	Species	*Potyvirus aconiti*	Aconitum virus 2	MZ389235
New taxon	Species	*Potyvirus puerariae*	kudzu chlorotic ring blotch virus	OQ148665
New taxon	Species	*Potyvirus alilii*	lily virus A	OR879085
New taxon	Species	*Potyvirus parisflavitessellati*	Paris yunnanensis mosaic chlorotic virus	ON871824
New taxon	Species	*Potyvirus catharanthiflavitessellati*	periwinkle mild yellow mosaic virus	PP382205
New taxon	Species	*Potyvirus polygonatimaculae*	Polygonatum kingianum mottle virus	ON428226
New taxon	Species	*Potyvirus crocitessellati*	saffron yellow mosaic virus	OK632024
New taxon	Species	*Potyvirus galanthi*	snowdrop virus Y	OP871788
New taxon	Genus	*Phragmivirus*		
New taxon	Species	*Phragmivirus phragmii*	common reed chlorotic stripe virus	KY612317
New taxon	Species	*Phragmivirus spartinae*	Spartina mottle virus	MN788417

*Source/full text: https://ictv.global/ictv/proposals/2024.012P.Potyviridae_1ng_10nsp.zip.

## 2024.013P.Secoviridae_1ng_2nsg_34nsp

**Title:** Create a new genus, two new subgenera and 34 new species in the family *Secoviridae* (*Picornavirales*)

**Authors:** Fuchs M. (mf13@cornell.edu), Hily J.-M., Petrzik K., Sanfaçon H., Stewart L., Thompson J.R., Van der Vlugt R.A.A., Wetzel T.

**Summary**

**Taxonomic rank(s) affected**
Genus, subgenus, species
**Description of current taxonomy**
The recognition of new virus species is based on demarcation criteria in the family *Secoviridae* of <75% amino acid sequence identity in the coat protein (CP)(s) and/or <80% amino acid sequence identity in the conserved protease (Pro)-polymerase (Pol) region (from the protease CG motif to the polymerase GDD motif) and/or distinct plant hosts and biological properties.
**Proposed taxonomic change(s)**
Create one (1) new genus (*Mersevirus*), two (2) new subgenera in the genus *Waikavirus* (*Ritunrivirus* and *Actinidivirus*), create two (2) new species in the genus *Fabavirus* (*Fabavirus betavitis* and *Fabavirus cirsii*), create four (4) new species in the new genus *Mersevirus* (*Mersevirus mercurialis*, *Mersevirus paris*, *Mersevirus boehmeriae* and *Mersevirus jujubae*), create two (2) new species in the genus *Nepovirus* (*Nepovirus betaparis* and *Nepovirus mirae*), create three (3) new species in the genus *Sadwavirus* (*Sadwavirus cattleyae*, *Sadwavirus gymnemae* and *Sadwavirus chrysanthemi*), create three (3) new species in the genus *Torradovirus* (*Torradovirus physalis*, *Torradovirus nanorugosum* and *Torradovirus arctii*) and create 20 new species in the genus *Waikavirus* (*Waikavirus ajugae*, *Waikavirus anacycli*, *Waikavirus betacamelliae*, *Waikavirus eleocharis*, *Waikavirus hirtae*, *Waikavirus juglandis*, *Waikavirus ligustici*, *Waikavirus mertensiae*, *Waikavirus populi*, *Waikavirus pedicularis*, *Waikavirus primulae*, *Waikavirus querci*, *Waikavirus ranunculi*, *Waikavirus thymi*, *Waikavirus trifoccidentale*, *Waikavirus thapsiae*, *Waikavirus violae*, *Waikavirus carotae*, *Waikavirus celtis* and *Waikavirus pittospori*).
**Justification**
The proposed new genus *Mersevirus* is based on the distinct genome organization of *Mersevirus mercurialis*, *Mersevirus paris*, *Mersevirus boehmeriae* and *Mersevirus jujubae* with a Ham1 domain with predicted inosine triphosphate pyrophosphatase activity at the C-terminus of the RNA-directed RNA polymerase – a feature unique among members of the family *Secoviridae* – and a grouping on a monophyletic clade of the amino acid sequence of the CPs and conserved Pro-Pol region. The proposed new subgenus *Ritunrivirus* is based on a statistically supported single lineage of 22 distinct species in the genus *Waikavirus* defined by the amino acid sequence of the combined three CPs and the conserved Pro-Pol region. The proposed new subgenus *Actinidivirus* is based on a statistically supported single lineage of 16 distinct species in the genus *Waikavirus* defined by the amino acid sequence of the combined 3 CPs and conserved Pro-Pol region. The creation of the proposed new 34 species is based on <75% amino acid sequence identity in the CP(s) and/or <80% amino acid sequence identity in the conserved Pro-Pol region (from the protease CG motif to the polymerase GDD motif) compared with members of classified species of the family *Secoviridae*.
**Submitted:** 10/06/24; **Revised**: 11/10/24

**Table 13** *Secoviridae*, 37 new taxa*

**Table:** 

Operation	Rank	New taxon name	Virus name	Exemplar
New taxon	Genus	*Mersevirus*		
New taxon	Species	*Mersevirus boehmeriae*	Boehmeria nivea secovirus	BK061322; BK061323
New taxon	Species	*Mersevirus jujubae*	jujube-associated virus 1	MT375548; MT375547
New taxon	Species	*Mersevirus mercurialis*	Mercurialis secovirus 1	OR544055; OR544056
New taxon	Species	*Mersevirus paris*	Paris polyphylla secovirus 2	BK061330; BK061331
New taxon	Subgenus	*Actinidivirus*		
New taxon	Species	*Waikavirus betacamelliae*	Camellia virus B	BK062984
New taxon	Species	*Waikavirus carotae*	carrot psyllid-borne associated virus	OM801008
New taxon	Species	*Waikavirus celtis*	hackberry virus A	OP533794
New taxon	Species	*Waikavirus hirtae*	Ficus hirta waikavirus	BK062987
New taxon	Species	*Waikavirus juglandis*	Juglans nigra waikavirus	BK062989
New taxon	Species	*Waikavirus pittospori*	Pittosporum tobira virus	OR659471
New taxon	Species	*Waikavirus populi*	Populus alba waikavirus	BK062992
New taxon	Species	*Waikavirus querci*	Quercus robur waikavirus	BK062996
New taxon	Species	*Waikavirus trifoccidentale*	Trifolium occidentale waikavirus	BK063000
New taxon	Subgenus	*Ritunrivirus*		
New taxon	Species	*Waikavirus ajugae*	Ajuga reptans waikavirus	BK062980
New taxon	Species	*Waikavirus anacycli*	Anacyclus depressus waikavirus	BK062979
New taxon	Species	*Waikavirus eleocharis*	Eleocharis dulcis waikavirus	BK062986
New taxon	Species	*Waikavirus ligustici*	Ligusticum chuanxiong waikavirus	BK062990
New taxon	Species	*Waikavirus mertensiae*	Mertensia paniculata waikavirus	BK062991
New taxon	Species	*Waikavirus pedicularis*	Pedicularis rex waikavirus	BK062993
New taxon	Species	*Waikavirus primulae*	Primula vulgaris waikavirus	BK062995
New taxon	Species	*Waikavirus ranunculi*	Ranunculus cantoniensis waikavirus	BK062997
New taxon	Species	*Waikavirus thapsiae*	Thapsia villosa waikavirus	BK063001
New taxon	Species	*Waikavirus thymi*	Thymus vulgaris waikavirus	BK062999
New taxon	Species	*Waikavirus violae*	Viola inconspicua waikavirus	BK063002
New taxon	Species	*Fabavirus betavitis*	grapevine secovirus	OR947508; OR947509
New taxon	Species	*Fabavirus cirsii*	Cirsium virus A	OP794357; OP794358
New taxon	Species	*Nepovirus betaparis*	Paris polyphylla secovirus 1	BK061328; BK061329
New taxon	Species	*Nepovirus mirae*	Prunus mira virus A	BK064709; BK064710
New taxon	Species	*Sadwavirus cattleyae*	Cattleya purple ringspot virus	OR439368; OR439369
New taxon	Species	*Sadwavirus gymnemae*	Gymnema sylvestre virus 1	BK062888; BK062889
New taxon	Species	*Sadwavirus chysanthemi*	chrysanthemum sadwavirus	OR413567; OR413568
New taxon	Species	*Torradovirus arctii*	burdock mosaic virus	OQ087134; OQ087135
New taxon	Species	*Torradovirus nanorugosum*	potato rugose stunting virus	ON871623; ON871624
New taxon	Species	*Torradovirus physalis*	Physalis torrado virus	MZ357183; MZ357184

**Table 14** *Secoviridae*, 13 move taxa*

**Table:** 

Operation	Rank	Taxon name	Old parent taxon	New parent taxon
Move taxon	Species	*Waikavirus actinidiae*	*Waikavirus*	Subgenus *Actinidivirus*
Move taxon	Species	*Waikavirus camelliae*	*Waikavirus*	Subgenus *Actinidivirus*
Move taxon	Species	*Waikavirus diospyri*	*Waikavirus*	Subgenus *Actinidivirus*
Move taxon	Species	*Waikavirus liegense*	*Waikavirus*	Subgenus *Actinidivirus*
Move taxon	Species	*Waikavirus rhododendri*	*Waikavirus*	Subgenus *Actinidivirus*
Move taxon	Species	*Waikavirus brassicae*	*Waikavirus*	Subgenus *Ritunrivirus*
Move taxon	Species	*Waikavirus campanulae*	*Waikavirus*	Subgenus *Ritunrivirus*
Move taxon	Species	*Waikavirus lactucae*	*Waikavirus*	Subgenus *Ritunrivirus*
Move taxon	Species	*Waikavirus oryzae*	*Waikavirus*	Subgenus *Ritunrivirus*
Move taxon	Species	*Waikavirus ribesnigri*	*Waikavirus*	Subgenus *Ritunrivirus*
Move taxon	Species	*Waikavirus rosae*	*Waikavirus*	Subgenus *Ritunrivirus*
Move taxon	Species	*Waikavirus trifolii*	*Waikavirus*	Subgenus *Ritunrivirus*
Move taxon	Species	*Waikavirus zeae*	*Waikavirus*	Subgenus *Ritunrivirus*

*Source/full text: https://ictv.global/ictv/proposals/2024.013P.Secoviridae_1ng_2nsg_34nsp.zip.

## 2024.014P.Rhabdoviridae_2nsp

**Title:** Create one new species in the genus *Alphanucleorhabdovirus* and one species in the genus *Betanucleorhabdovirus*, subfamily *Betarhabdovirinae* (*Mononegavirales*: *Rhabdoviridae*)

**Authors:** Bejerman N. (bejerman.nicolas@inta.gob.ar), Debat H., Dietzgen R., Freitas-Astua J., Kondo H., Ramos-Gonzalez P., Whitfield A., Walker P.

**Summary**

**Taxonomic rank(s) affected**
Species
**Description of current taxonomy**
Viruses classified in the genera *Alphanucleorhabdovirus* and *Betanucleorhabdovirus* infect a wide range of plants, and the assignment of viruses to these genera is based on the placement of the viruses on maximum likelihood trees inferred from complete L protein sequences.
**Proposed taxonomic change(s)**
Add one (1) new species to the genus *Alphanucleorhabdovirus* (*Alphanucleorhabdovirus babaci*) and one (1) new species in the genus *Betanucleorhabdovirus* (*Betanucleorhabdovirus paridis*).
**Justification**
Two novel rhabdoviruses were identified in babaco [17] and *Paris polyphylla* [18]. The characterization of both viruses showed that the babaco-associated virus should be classified as a novel species within the genus *Alphanucleorhabdovirus*, while the *P. polyphylla-*associated virus should be classified as a novel species within the genus *Betanucleorhabdovirus*.
**Submitted:** 10/06/24

**Table 15** *Rhabdoviridae*, 2 new taxa*

**Table:** 

Operation	Rank	New taxon name	Virus name	Exemplar
New taxon	Species	*Alphanucleorhabdovirus babaci*	babaco nucleorhabdovirus 1	OQ256237
New taxon	Species	*Betanucleorhabdovirus paridis*	Paris yunnanensis rhabdovirus 1	OL439478

*Source/full text: https://ictv.global/ictv/proposals/2024.014P.Rhabdoviridae_2nsp.zip.

## 2024.015P.Rhabdoviridae_Cytorhabdovirus_splitgen

**Title:** Abolish one genus and create three new genera to include 98 new species in the subfamily *Betarhabdovirinae* (*Mononegavirales*: *Rhabdoviridae*)

**Authors:** Bejerman N. (bejerman.nicolas@inta.gob.ar), Debat H., Dietzgen R., Freitas-Astua J., Kondo H., Ramos-Gonzalez P., Whitfield A., Walker P.

**Summary**

**Taxonomic rank(s) affected**
Genus and species
**Description of current taxonomy**
Viruses classified in the genus *Cytorhabdovirus* infect a wide range of plants, and the assignment of viruses to this genus is based on the placement of the viruses on maximum likelihood trees inferred from complete L protein sequences.
**Proposed taxonomic change(s)**
Abolish the genus *Cytorhabdovirus* and add three (3) new genera (*Alphacytorhabdovirus*, *Betacytorhabdovirus* and *Gammacytorhabdovirus*), including 98 new species in the subfamily *Betarhabdovirinae* (*Mononegavirales*: *Rhabdoviridae*). Abolish four (4) *Cytorhabdovirus* species and reassign the remaining species to the new genera.
**Justification**
Recently, 98 new putative cytorhabdoviruses were discovered. The phylogenetic relationships of the now significantly expanded number of known cytorhabdoviruses provide support for splitting the genus *Cytorhabdovirus* into three genera that represent distinct evolutionary lineages to be named *Alphacytorhabdovirus*, *Betacytorhabdovirus* and *Gammacytorhabdovirus*. Also, it is proposed that four *Cytorhabdovirus* species be abolished based on the lack of sequence data for them.
**Submitted:** 10/06/24; **Revised:** 03/10/24

**Table 16** *Rhabdoviridae*, 101 new taxa*. Table too large, see supplementary information sheet supp_info_tab_16

**Table 17** *Rhabdoviridae,* 51 move; rename taxa*. Table too large, see supplementary information sheet supp_info_tab_17

**Table 18** *Rhabdoviridae*, 4 abolish taxa*

**Table:** 

Operation	Rank	Abolished taxon name
Abolish taxon	Species	*Cytorhabdovirus brassicae*
Abolish taxon	Species	*Cytorhabdovirus festucae*
Abolish taxon	Species	*Cytorhabdovirus sonchi*
Abolish taxon	Species	*Cytorhabdovirus tritici*

*Source/full text: https://ictv.global/ictv/proposals/2024.015P.Rhabdoviridae_Cytorhabdovirus_splitgen.zip.

## 2024.016P.Rhabdoviridae_1ngen_5nsp

**Title:** Create one new genus to include five new species in the subfamily *Betarhabdovirinae* (*Mononegavirales*: *Rhabdoviridae*)

**Authors:** Bejerman N. (bejerman.nicolas@inta.gob.ar), Debat H., Dietzgen R.G., Freitas-Astua J., Kondo H., Ramos-Gonzalez P., Whitfield A., Walker P.

**Summary**

**Taxonomic rank(s) affected**
Genus and species
**Description of current taxonomy**
Almost all viruses classified in the subfamily *Betarhabdovirinae*, family *Rhabdoviridae*, are unsegmented, but plant-associated rhabdoviruses with bi-segmented genomes have also been identified and included in the genera *Varicosavirus* and *Dichorhavirus* within the subfamily *Betarhabdovirinae*. The assignment of viruses to these genera is based on the placement of the viruses on maximum likelihood trees inferred from complete L protein sequences.
**Proposed taxonomic change(s)**
Add one (1) new genus to include five (5) new species in the subfamily *Betarhabdovirinae*, family *Rhabdoviridae*. These new species (*Trirhavirus alni*, *Trirhavirus chrysanthemi*, *Trirhavirus erysimi*, *Trirhavirus medicagonis* and *Trirhavirus picridis*) are placed in a new genus named *Trirhavirus*.
**Justification**
Five novel rhabdoviruses were identified in *Alnus rubra*, *Chrysanthemum morifolium*, *Erysimum nevadense*, *Medicago sativa* and *Picris echioides* [19]. Unexpectedly, these five viruses have tri-segmented genomes, which represent the first tri-segmented genomes among rhabdoviruses. The characterization of these five viruses showed they should be classified as members of novel species within a novel genus within the subfamily *Betarhabdovirinae*, family *Rhabdoviridae*, for which the name *Trirhavirus* is proposed [19].
**Submitted:** 10/06/24

**Table 19** *Rhabdoviridae*, 6 new taxa*

**Table:** 

Operation	Rank	New taxon name	Virus name	Exemplar
New taxon	Genus	*Trirhavirus*		
New taxon	Species	*Trirhavirus alni*	Alnus trirhavirus 1	BK064247; BK064248; BK064249
New taxon	Species	*Trirhavirus chrysanthemi*	chrysanthemum trirhavirus 1	BK064250; BK064251; BK064252
New taxon	Species	*Trirhavirus erysimi*	Erysimum trirhavirus 1	BK064253; BK064254; BK064255
New taxon	Species	*Trirhavirus medicagonis*	Medicago trirhavirus	BK064256; BK064257; BK064258
New taxon	Species	*Trirhavirus picridis*	Picris trirhavirus 1	BK064259; BK064260; BK064261

*Source/full text: https://ictv.global/ictv/proposals/2024.016P.Rhabdoviridae_1ngen_5nsp.zip.

## 2024.017P.Tombusviridae_abolishsp

**Title:** Abolish five unassigned species in the family *Tombusviridae*

**Authors:** Scheets K. (kay.scheets@okstate.edu), Hernández C., Jordan R., Miller W.A., Prigigallo M.I., Rubino L.

**Summary**

**Taxonomic rank(s) affected**
Species in the family *Tombusviridae*
**Description of current taxonomy**
*Ahlum waterborne virus*, *Bean mild mosaic virus*, *Chenopodium necrosis virus*, *Cucumber soil-borne virus* and *Weddel waterborne virus* are currently classified as unassigned species in the family *Tombusviridae*.
**Proposed taxonomic change(s)**
Abolish five (5) species.
**Justification**
The proposed abolishment of these species is based on the lack of sequence data for them.
**Submitted:** 21/06/24

**Table 20** *Tombusviridae*, 5 abolish taxa*

**Table:** 

Operation	Rank	Abolished taxon name
Abolish taxon	Species	*Ahlum waterborne virus*
Abolish taxon	Species	*Bean mild mosaic virus*
Abolish taxon	Species	*Chenopodium necrosis virus*
Abolish taxon	Species	*Cucumber soil-borne virus*
Abolish taxon	Species	*Weddel waterborne virus*

*Source/full text: https://ictv.global/ictv/proposals/2024.017P.Tombusviridae_abolishsp.zip.

## 2024.018P.Tombusviridae_1nsp

**Title:** Create one new species in the genus *Machlomovirus* (*Tolivirales*: *Tombusviridae*)

**Authors:** Maclot F., Massart S. (sebastien.massart@uliege.be)

**Summary**

**Taxonomic rank** **(s) affected**
Genus *Machlomovirus* (*Tolivirales*: *Tombusviridae*)
**Description of current taxonomy**
One virus species, *Machlomovirus zeae*, is currently described within the genus *Machlomovirus*.
**Proposed taxonomic change(s)**
Add a second species (*Machlomovirus liegense*) to the genus *Machlomovirus* to accommodate a recently identified virus in the wild grass common bent (*Agrostis capillaris*), tentatively named Poaceae Liege machlomovirus.
**Justification**
Analysis of Poaceae Liege machlomovirus genomic structure and phylogenetic analyses of its complete sequence and specific genes (polymerase and coat protein) placed this virus as a member of a novel species in the genus *Machlomovirus*.
**Submitted:** 21/06/24

**Table 21** *Tombusviridae*, 1 new taxon*

**Table:** 

Operation	Rank	New taxon name	Virus name	Exemplar
New taxon	Species	*Machlomovirus liegense*	Poaceae Liege machlomovirus	ON137711

*Source/full text: https://ictv.global/ictv/proposals/2024.018P.Tombusviridae_1nsp.zip.

## 2024.019P.Closteroviridae_1nsp

**Title:** Create one new species in the genus *Velarivirus* (order *Martellivirales*, family *Closteroviridae*)

**Authors:** Fontdevila N., Massart S. (sebastien.massart@uliege.be)

**Summary**

**Taxonomic rank(s) affected**
Genus *Velarivirus* (order *Martellivirales*, family *Closteroviridae*)
**Description of current taxonomy**
The family *Closteroviridae* comprises plant viruses with long, filamentous particles (650–2,200 nm in length) and large positive-sense RNA genomes (mono-, bi- or tripartite). There are 57 recognized species in the family, classified in 1 of the 7 existing genera (*Ampelovirus*, *Bluvavirus*, *Closterovirus*, *Crinivirus*, *Menthavirus*, *Olivavirus* and *Velarivirus*). Within the genus *Velarivirus*, there are currently eight recognized species.
**Proposed taxonomic change(s)**
The authors propose adding a ninth species in the genus *Velarivirus*, named *Velarivirus gembloutense*, to classify the recently identified virus Pyrus virus A in pear trees (*Pyrus communis* L.).
**Justification**
Analysis of the genomic structure of Pyrus virus A and subsequent phylogenetic analyses of the specific HSP70h gene placed this virus as a member of a novel species of the genus *Velarivirus* within the family *Closteroviridae*.
**Submitted:** 21/06/24; **Revised:** 07/10/24

**Table 22** *Closteroviridae*, 1 new taxon*

**Table:** 

Operation	Rank	New taxon name	Virus name	Exemplar
New taxon	Species	*Velarivirus gembloutense*	Pyrus virus A	OR887735

*Source/full text: https://ictv.global/ictv/proposals/2024.019P.Closteroviridae_1nsp.zip.

## 2024.021P.Riboviria_1nord

**Title:** Create one new unassigned order in realm *Riboviria*, and three new families for four currently unassigned genera of plant satellite viruses

**Authors:** Krupovic M. (mart.krupovic@pasteur.fr), Fischer M.G., Kuhn J.H.

**Summary**

**Taxonomic rank(s) affected**
Species, genus, family
**Description of current taxonomy**
*Riboviria*: unassigned family *Sarthroviridae* and genera *Albetovirus*, *Aumaivirus*, *Papanivirus* and *Virtovirus*.
**Proposed taxonomic change(s)**
*Riboviria*: *Tombendovirales* to include family *Sarthroviridae* and two (2) new families, *Pamosaviridae* (*Papanivirus*) and *Tomosaviridae* (*Virtovirus*); *Riboviria*: *Tonesaviridae* (*Albetovirus*, *Aumaivirus*); renaming of all species in the four genera to fulfil the ICTV’s binomial naming mandate.
**Justification**
Structural comparison of the satellite virus capsid proteins indicates that these viruses are not monophyletic and form two distinct assemblages.
**Submitted:** 21/06/24

**Table 23** *Riboviria*, 6 rename taxa*

**Table:** 

Operation	Rank	New taxon name	Previous taxon name
Rename taxon	Species	*Papanivirus panici*	*Panicum papanivirus 1*
Rename taxon	Species	*Virtovirus tabaci*	*Tobacco virtovirus 1*
Rename taxon	Species	*Albetovirus alphatabaci*	*Tobacco albetovirus 1*
Rename taxon	Species	*Albetovirus betatabaci*	*Tobacco albetovirus 2*
Rename taxon	Species	*Albetovirus gammatabaci*	*Tobacco albetovirus 3*
Rename taxon	Species	*Aumaivirus maidis*	*Maize aumaivirus 1*

**Table 24** *Riboviria*, 5 move taxa*

**Table:** 

Operation	Rank	Taxon name	New parent taxon	Old parent taxon
Move taxon	Family	*Sarthroviridae*	*Tombendovirales*	*Riboviria*
Move taxon	Genus	*Papanivirus*	*Pamosaviridae*	*Riboviria*
Move taxon	Genus	*Virtovirus*	*Tomosaviridae*	*Riboviria*
Move taxon	Genus	*Albetovirus*	*Tonesaviridae*	*Riboviria*
Move taxon	Genus	*Aumaivirus*	*Tonesaviridae*	*Riboviria*

**Table 25** *Riboviria*, 4 new taxa*

**Table:** 

Operation	Rank	New taxon name
New taxon	Order	*Tombendovirales*
New taxon	Family	*Pamosaviridae*
New taxon	Family	*Tomosaviridae*
New taxon	Family	*Tonesaviridae*

*Source/full text: https://ictv.global/ictv/proposals/2024.021P.Riboviria_1nord.zip.

## 2024.022P.Betaflexiviridae_abolishsp

**Title:** Abolish one unassigned species in the family *Betaflexiviridae*

**Authors:** Nagata T. (tatsuya@unb.br), Blouin A., Candresse T., Cao M., Cho W.K., Constable F., Sabanadzovic S., Saldarelli P., Tzanetakis I., Villamor D.

**Summary**

**Taxonomic rank(s) affected**
Species in the family *Betaflexiviridae*
**Description of current taxonomy**
*Banana virus X* is currently classified as an unassigned species in the family *Betaflexiviridae*.
**Proposed taxonomic change(s)**
Abolish one (1) species.
**Justification**
The proposed abolishment of this betaflexivirid species is based on the lack of Rep sequence data.
**Submitted:** 30/06/24

**Table 26** *Betaflexiviridae*, 1 abolish taxon*

**Table:** 

Operation	Rank	Abolished taxon name
Abolish taxon	Species	*Banana virus X*

*Source/full text: https://ictv.global/ictv/proposals/2024.022P.Betaflexiviridae_abolishsp.zip.

## 2024.023P.Solemoviridae_rename_sp

**Title:** Rename two species in the genus *Sobemovirus* (family *Solemoviridae*)

**Authors:** Sõmera M. (merike.somera@taltech.ee), Fargette D., Filardo F., Ghafari M., Hebrard E., Sarmiento C., Thomas J.E.

**Summary**

**Taxonomic rank(s) affected**
Species
**Description of current taxonomy**
*Riboviria*: *Orthornavirae*: *Pisuviricota*: *Pisoniviricetes*: *Sobelivirales*: *Solemoviridae*: *Sobemovirus*: *Cocksfoot mottle virus*. *Riboviria*: *Orthornavirae*: *Pisuviricota*: *Pisoniviricetes*: *Sobelivirales*: *Solemoviridae*: *Sobemovirus*: *Sobemovirus smamv*.
**Proposed taxonomic change(s)**
Renaming of *Cocksfoot mottle virus* and *Sobemovirus smamv*.
**Justification**
Renaming of *Cocksfoot mottle virus* to fulfil the ICTV’s binomial naming mandate. Renaming of *Sobemovirus smamv* for consistency with other species in the family *Solemoviridae*.
**Submitted:** 28/06/24; **Revised:** 07/10/24

**Table 27** *Solemoviridae*, 2 rename taxa*

**Table:** 

Operation	Rank	New taxon name	Previous taxon name
Rename taxon	Species	*Sobemovirus CFMV*	*Cocksfoot mottle virus*
Rename taxon	Species	*Sobemovirus SMAMV*	*Sobemovirus smamv*

*Source/full text: https://ictv.global/ictv/proposals/2024.023P.Solemoviridae_rename_sp.zip.

## 2024.024P.Tymoviridae_abolish_sp

**Title:** Abolish two unassigned species in the family *Tymoviridae*

**Authors:** Hammond R. (rose.hammond@usda.gov), Abrahamian P., Bejerman N., Mollov D., Nagata T., Sabanadzovic S.

**Summary**

**Taxonomic rank(s) affected**
Species in the family *Tymoviridae*
**Description of current taxonomy**
*Bombyx mori latent virus* and *Poinsettia mosaic virus* are currently classified as unassigned species in the family *Tymoviridae*.
**Proposed taxonomic change(s)**
Abolish two (2) species.
**Justification**
*Bombyx mori latent virus* and *Poinsettia mosaic virus* are unassigned species in the family *Tymoviridae* which cannot be assigned to a genus because of the lack of genome sequence. Therefore, it is not possible to comply with the ICTV mandate of a binomial format for virus species and it is proposed that these tymovirid species be abolished.
**Submitted:** 14/07/24

**Table 28** *Tymoviridae*, 2 abolish taxa*

**Table:** 

Operation	Rank	Abolished taxon name
Abolish taxon	Species	*Bombyx mori latent virus*
Abolish taxon	Species	*Poinsettia mosaic virus*

*Source/full text: https://ictv.global/ictv/proposals/2024.024P.Tymoviridae_abolish_sp.zip.

## Supplementary material

10.1099/jgv.0.002114 Supplementary Material 1.

## Abbreviations

CP: coat protein
ICTV: International Committee on Taxonomy of Viruses
Pol: polymerase
Pro: protease
